# Concomitant anti-CGRP and immunomodulatory treatments in patients with migraine: towards integrated management strategies

**DOI:** 10.1007/s00415-025-13177-y

**Published:** 2025-06-03

**Authors:** María Clara García-Castillo, Álvaro Sierra-Mencía, Edoardo Caronna, Daniel Toledo-Alfocea, Alex Jaimes, Saray Urtiaga, Javier Casas-Limón, Albert Muñoz-Vendrell, Sonia Santos-Lasaosa, Valvanuz García Martín, Guillermo Martín Ávila, Marcos Polanco, Maria Dolores Villar-Martínez, Cristina Trevino-Peinado, Laura Rubio-Flores, Antonio Sánchez-Soblechero, Leonardo Portocarrero Sánchez, Elisa Luque-Buzo, Alberto Lozano-Ros, Ana Beatriz Gago-Veiga, Javier Díaz-De-Terán, Andrea Recio García, Javiera Canales Rodríguez, Andrea Gómez García, Marta González Salaices, Sergio Campoy, Ane Mínguez-Olaondo, Stefania Maniataki, Vicente González-Quintanilla, Jesús Porta-Etessam, María-Luz Cuadrado, Ángel Luis Guerrero Peral, Patricia Pozo-Rosich, Jaime Rodríguez-Vico, Mariano Huerta-Villanueva, Julio Pascual, Peter J. Goadsby, Alicia Gonzalez-Martinez

**Affiliations:** 1 Facultad de Medicina, Universidad Autónoma de Madrid (UAM), Madrid, Spain; 2 Hospital Universitario de la Princesa, Madrid, Spain; 3 Headache Unit, Neurology Department, Hospital Clínico Universitario de Valladolid, Valladolid, Spain; 4 Headache Clinic, Neurology Department, Hospital Universitari Vall d’Hebron, Barcelona, Spain; 5 Headache and Neurological Pain Research Group, Vall d’Hebron Research Institute, Barcelona, Spain; 6 Servicio de Neurología, Hospital 12 Octubre, Madrid, Spain; 7 Neurology Department, Fundación Jiménez Díaz University Hospital, Madrid, Spain; 8 Neurology Department, Hospital de Torrejón, Madrid, Spain; 9 Headache Unit, Neurology Department, Hospital Universitario Fundación Alcorcón, Alcorcón, Spain; 10 Headache Unit, Neurology Department, Hospital Universitari de Bellvitge-IDIBELL, Universitat de Barcelona, L’Hospitalet de Llobregat, Barcelona, Spain; 11 Neurology Department, Hospital Universitario Lozano Blesa. IIS Aragon, University of Zaragoza, Zaragoza, Spain; 12 Instituto de Investigación Sanitaria (IIS) Biogipuzkoa, San Sebastián, Spain; 13 Neurology Department, Hospital de Getafe, Getafe, Spain; 14 Neurology Department, Marqués de Valdecilla University Hospital, Santander, Spain; 15 NIHR King’s Clinical Research Facility and Wolfson SPaRC King’s College London, London, UK; 16 Headache Clinic, Neurology Department, Severo Ochoa University Hospital, Leganés, Spain; 17 Headaches, Craniofacial Pain and Neurological Pain Unit, Vithas Hospitals Group, Vithas Clinical Neuroscience Institute, La Milagrosa, Aravaca & Arturo Soria University Hospitals, Madrid, Spain; 18 Neurology Department, Hospital General Universitario Gregorio Marañón, Madrid, Spain; 19 Neurology Department, Hospital Universitario de la Paz, Madrid, Spain; 20 Hospital Universitario de la Princesa, Instituto de Investigación Sanitaria Princesa (IIS-Princesa), Universidad Autónoma de Madrid, Madrid, Spain; 21 Hospital San Martín de Quillota, Quillota, Chile; 22 Headache Unit, Hospital Universitari de Bellvitge & Hospital de Viladecans - IDIBELL, Universitat de Barcelona, Barcelona, Spain; 23 Neurology Department, Hospital Universitario Donostia-Osakidetza, Neuroscience Area, Biogipuzkoa Health Institute, Donostia, Spain; 24 Department of Medicine and Department of Physical Therapy, Faculty of Health Sciences, University of Deusto, Bilbao, San Sebastian, Spain; 25 School of Medicine, Department of Neurology, Hospital Clínico San Carlos, Universidad Complutense de Madrid, Madrid, Spain; 26 Departamento de Medicina, Universidad de Valladolid, Valladolid, Spain; 27 Department of Neurology, King’s College Hospital, London, UK; 28 Department of Neurology, University of California, Los Angeles, CA USA; 29 Neurology and Immunology Department, Hospital Universitario de La Princesa, Calle Diego de León, 62, 28006 Madrid, Spain

**Keywords:** Immunity, CGRP, Monoclonal antibodies, Combination therapy, Immunosuppression, Neuroinflammation

## Abstract

**Background:**

Preclinical evidence supports the immunoregulatory role of calcitonin gene-related peptide (CGRP) in migraine pathophysiology. The increasing use of anti-CGRP therapies in patients with migraine and other comorbidities raises the question whether the potential use of anti-CGRP monoclonal antibodies (CGRP-mAbs) therapies in combination with other immunological therapies is effective and safe.

**Methods:**

This multicenter study included patients with migraine receiving CGRP-mAbs combined with immunosuppressive and immunomodulatory treatments. Clinical and demographic data, treatment history, laboratory markers and treatment-emergent adverse events (TEAEs) were analyzed. Effectiveness outcomes included the change in monthly migraine days (MMD) and monthly headache days (MHD) at 3, 6, 9 and 12 months, alongside the > 50% response rate. Moreover, autoimmune disease progression was also evaluated. We explored differences between patients with and without autoimmune disease activation.

**Results:**

Among 89 patients, there were 80 (90%) females with a mean age of 50 years (SD: 11), who had a high prevalence of psychiatric comorbidities (anxiety 44%, depression 49%) and medication overuse (68%). Patients receiving immunological treatments experienced significant reductions in MMD and MHD, with MMD decreasing from 16 (SD: 7) at baseline to 9 (SD: 8) at 6 months, and MHD dropping from 23 (SD: 8) to 17 (SD: 11). A 50% response in MMD was achieved by 46% at 6 months. TEAEs were reported in 28%, most commonly constipation (16%) and dizziness (9%).

**Conclusions:**

CGRP-mAbs therapies combined with immunological treatments appear effective and safe in patients with autoimmune diseases. Larger prospective studies are necessary to confirm these findings and optimize management strategies.

**Supplementary Information:**

The online version contains supplementary material available at 10.1007/s00415-025-13177-y.

## Background

Migraine is a highly prevalent and disabling neurological disorder, affecting millions worldwide and ranking among the leading causes of years lived with disability [1–4]. Recent research has highlighted an increased prevalence of migraine in patients with various chronic inflammatory diseases. For instance, a recent meta-analysis published in 2023 reported that the prevalence of migraine among patients with multiple sclerosis (MS) was 24% [5]. Similarly, emerging evidence supports a higher prevalence of migraine among patients with inflammatory bowel disease [6, 7]. This growing body of evidence underscores the need for studies that address relevant aspects for diagnosis and treatment management in patients with migraine and chronic inflammatory conditions.

The intricate pathophysiology of migraine, involving peripheral and central mechanisms, has driven advances in targeted therapies such as monoclonal antibodies against calcitonin gene-related peptide or its receptor (CGRP-mAbs) [8]. It has been speculated that calcitonin gene-related peptide (CGRP) is involved in intracranial vasodilation and the activation of neuro-inflammatory cascades through the trigeminovascular system in migraine pathophysiopathology [9], although the inflammatory mechanism has not been established [10]. CGRP-mAbs have emerged as a groundbreaking option for migraine prevention, both in patients with chronic and episodic forms of the disease [11], and also for special populations such as patients over 65 years old [12].

Autoimmune disorders, generally characterized by immune dysregulation and chronic inflammation, are frequently accompanied by headache and/or comorbid with migraine, likely due to shared pathophysiological mechanisms such as neuroinflammation, endothelial dysfunction, and altered immune responses [6, 7, 13, 14]. These findings highlight the potential need of using CGRP-mAbs in concomitance with other immunological treatments. Moreover, a potential role of CGRP as a regulator of the immune system has been hypothesized based on case series reporting inflammatory complications in eight patients treated with CGRP-mAbs [15]. This interplay underscores the need to evaluate further whether immunomodulatory therapies impact response and tolerability to CGRP-mAbs in patients with migraine. Understanding this relationship can provide critical insights into optimizing therapeutic strategies, minimizing potential drug–drug interactions, and improving outcomes for a unique subset of patients with overlapping neurological and autoimmune conditions. It is noteworthy that CGRP-mAbAs have been engineered in the Fc region as immunopharmacotherapies, which is likely to influence interactions with not immunomodulatory therapies. CGRP-mAbs have undergone glycoengineering [16], such as is the case for eptinezumab [17], or had their FC region amino acid sequences altered, such as with fremanezumab [18]. Furthermore, these investigations have significant implications for clinical practice. Establishing evidence-based guidelines for managing migraine in patients receiving immunological treatments can enhance personalized care and inform multidisciplinary collaboration between neurologists, rheumatologists, and other specialists. This article seeks to characterize the profiles of migraine patients receiving both CGRP-mAbs therapies and concomitant immunological treatments, providing a foundation for clinical recommendations and future research.

Our principal objective is to describe the effectiveness and tolerability of the use of CGRP-mAbs immunological drugs. Secondary objectives include (1) to describe the percentage and characteristics of patients with autoimmune disease activation and (2) to explore the potential differences between patients with and without autoimmune disease activation during treatment with anti-CGRP-mAbs.

## Material and methods

### Study design

This is a retrospective multicenter study involving patients treated with CGRP-mAbs and immunological therapies. These patients were included in prospective cohorts of migraine cases attended at Headache Units/Headache Clinics in Spain and the United Kingdom. In accordance with national clinical guidelines and reimbursement criteria, patients initiating anti-CGRP monoclonal antibody therapy had experienced eight or more monthly migraine days and had previously undergone at least three adequate trials of preventive treatments over a minimum of 3 months. For patients with chronic migraine, this included treatment with onabotulinumtoxinA. At the time of initiating anti-CGRP therapy, patients were receiving concurrent treatments for their immunological disorders.

Inclusion criteria were (1) patients over 18 years old; (2) patients with migraine under CGRP-mAbs treatment; (3) presence of any of the following autoimmune disorders: neurological diseases (multiple sclerosis), rheumatological (arthritis, lupus, vasculitis), gastrointestinal (inflammatory bowel disease, autoimmune hepatitis), dermatological (psoriasis), and autoimmune-linked hypersensitivity syndromes (asthma, hereditary angioedema, chronic urticaria); (4) concomitant immunological treatment. Exclusion criteria were (1) not willing to participate in the study and (2) contraindications for the use of CGRP-mAbs.

### Data collection

Data were collected between May and October 2024 from CGRP-mAbs cohort databases at Headache Units and Clinics, including only patients meeting the inclusion criteria. The e-diaries included both electronic and paper-based formats, in line with routine clinical practice in each of the Headache Units. Approval for the study was obtained from the institutional ethics committee of Hospital Universitario de la Princesa (Number: 4563).

### Variables included in the study

The study included demographic and clinical characteristics such as sex, age, and age at migraine onset; vascular risk factors, including high blood pressure (HBP), dyslipidemia (DL), diabetes mellitus (DM), active smoking, and alcoholic consumption; psychological comorbidities such as anxiety, depression, and insomnia were also evaluated; migraine type, including chronic migraine (CM) and episodic migraine (EM), as well as migraine aura (MA); clinical characteristics such as time with migraine and time with CM; medication overuse, the number of prior preventive treatments and concomitant preventive therapies. We collected concomitant immunological treatments such as amino salicylates, immunosuppressants such as corticosteroids, antimetabolites (methotrexate, azathioprine, mercaptopurine, mycophenolate mofetil), calcineurin Inhibitors (tacrolimus), JAK Inhibitors (tofacitinib), Anti-CD20 (rituximab, ocrelizumab, ofatumumab), IFN or immunomodulators such as IL Inhibitors (IL-5: mepolizumab, reslizumab; IL-6: tocilizumab; IL-17: ixekizumab, ustekinumab), TNF Inhibitors (adalimumab, etanercept, infliximab, golimumab), B-cell modulators (belimumab), Anti-Integrin (abatacept), Anti-IgE (omalizumab), and CD52 modulators (cladribine) (hydroxychloroquine, montelukast, siponimod, dimethyl fumarate, glatiramer acetate, leflunomide). Response to treatment was documented including the reduction of number of monthly migraine days (MMD) and/or monthly headache days (MHD) after 3, 6, 9, 12 and 12 months and 50% response rate. Tolerability and safety, the presence of emerging adverse events (TEAEs), were noted. Presence and type of temporarily related autoimmune disease activation after CGRP-mAbs therapies start was recorded. All data were managed in a pseudonymized manner.

### Statistical analysis

The results obtained were analyzed using descriptive and analytical statistical techniques. A description of the effectiveness and safety of CGRP-mAbs drugs was made. Correlating studies of clinical and demographic variables were also performed. Measures of central tendency and dispersion were obtained for quantitative variables, as well as absolute and relative frequencies for qualitative variables. The type of distribution of quantitative variables was examined, and their alignment with a Gaussian distribution was assessed using the Kolmogorov–Smirnov test. For normally distributed data, parametric methods such as the Student’s *t*-test were employed to compare means between groups. For data that did not meet the normality assumptions, non-parametric methods like the Mann–Whitney *U* test were used instead. Chi-square or Fisher’s exact tests were applied for comparing categorical variables. Correlations between variables were calculated using Pearson’s correlation coefficient for parametric data or Spearman’s rank correlation for non-parametric data. Statistical analysis was conducted using the SPSS (version 16.0 for Windows) and R (version 1.4.1717)). We did not conduct a sample size calculation prior to the study which was based on available data. Patients with at least two effectiveness timepoints (baseline and follow-up) for migraine data and less than 20% missing data were included in the study. *P-*values presented are for a two-tailed test, and we considered *P* values < 0.05 as statistically significant.

## Results

### Baseline characteristics

89 patients were included in the study, with a mean age of 50 years (SD: 11), ranging from 21 to 79 years. The majority were women (90%). Vascular risk factors among the patients included HBP (12%), DL (16%), DM (3%), and active smoking (20%), while no patients reported alcohol consumption.

Comorbidities also included anxiety (44%), depression (49%) and insomnia (29%); chronic migraine (85%) and migraine aura (27%). The mean age of migraine onset was 22 years (SD: 10), and the mean duration of chronic migraine was 11 months (SD: 9). Medication overuse was observed in 68% of patients. Patients had a mean of 5 prior preventive treatments (SD: 3). At the initiation of anti-CGRP combined with immunomodulatory treatment, 37 patients (42%) were receiving other preventive migraine therapy. Of these, 33 patients (37%) were on antiepileptics, 32 (36%) on antidepressants, 15 (17%) on antihypertensives, and 16 (18%) were receiving onabotulinumtoxinA. In addition, 20% of patients were under corticosteroid treatment and the mean duration of CGRP-mAbs and immunological treatment was 17 months (SD: 16). Table 1 summarizes all baseline variables in the study group.

**Table 1:** Demographic and clinical characteristics of the patients included in the study

Variables	*n* = *89*
Age, years (SD), min–max	50 (11), 21–79
Sex, female (%)	80 (90%)
Autoimmune disease type, *n* (%)	
Neurological, *n* (%)	17
Rheumatological, *n* (%)	41
Gastrointestinal, *n* (%)	24
Dermatological, *n* (%)	4
Autoimmune-linked hypersensitivity syndromes, *n* (%)	4
Vascular risk factors	
HBP, *n* (%)	11 (12%)
DL, *n* (%)	14 (16%)
DM, *n* (%)	3 (3%)
Active smoking, *n* (%)	18 (20%)
Alcoholic consumption, *n* (%)	0 (0%)
Other comorbidities	
Anxiety, *n* (%)	39 (44%)
Depression, *n* (%)	44 (49%)
Insomnia, *n* (%)	26 (29%)
Age of migraine onset, mean (SD)	22 (10)
Chronic migraine, *n* (%)	76 (85%)
Migraine aura, *n* (%)	24 (27%)
Time with migraine, years (SD)	27 (12)
Time with chronic migraine, months (SD)	11 (9)
Medication overuse, *n* (%)	59 (68%)
Number of prior preventive treatments, mean (SD)	5 (3)
Anti-CGRP treatment type	
Erenumab, *n* (%)	17 (19%)
Galcanezumab,* n* (%)	28 (31%)
Fremanezumab,* n* (%)	44 (49%)
Other concomitant migraine preventive treatment, *n* (%)	37 (42%)
CGRP-mAbs plus immunomodulatory treatment duration, months (SD)	17 (16)

HBP, high blood pressure; DL, dyslipidemia; DM, diabetes mellitus; SD, standard deviation

### Laboratory inflammatory markers in patients under CGRP-mAbs and immunological treatments

At baseline, most patients being treated with CGRP-mAbs with immunological treatments exhibited normal levels for several inflammatory and hematological markers such as C-reactive protein (CRP), rheumatoid factor (RF), and erythrocyte sedimentation rates (VSG), and did not show lymphopenia. Yet, 11% of patients presented elevated CRP levels, 22% elevated RF levels and 14% elevated VSG at baseline. Regarding lymphopenia, 6% of patients had grade 1, while 2% experienced grade 2. Laboratory markers values at baseline are included in Table 2.

**Table 2:** Laboratory inflammatory markers in patients under CGRP-mAbs and immunomodulatory treatments

Laboratory markers	*n* = *64*
Baseline C-reactive protein	64/64 (100%)
Normal, *n* (%)	57 (89%)
Elevated,* n* (%)	7 (11%)
Baseline rheumatoid factor	51/64 (80%)
Normal, *n* (%)	40 (78%)
Elevated, *n* (%)	11 (22%)
Baseline VSG	56/64 (87%)
Normal, *n* (%)	48 (85%)
Elevated, *n* (%)	8 (14%)
Baseline lymphopenia	62/64 (97%)
No lymphopenia, *n* (%)	57 (92%)
Lymphopenia grade 1,* n* (%)	4 (6%)
Lymphopenia grade 2, *n* (%)	1 (2%)

VSG, erythrocyte sedimentation rate; Lymphopenia grade 1: 800–1,000 cells/μL, lymphopenia grade 2: 500–799 cells/μL

### Effectiveness in patients under CGRP-mAbs with immunomodulatory treatments

In patients with migraine treated with CGRP-mAbs in combination with immunological treatments, improvements were observed in MMD and MHD over a 12-month period. At baseline, the mean MMD was 16 (SD: 7), and the mean MHD was 23 (SD: 8). At 6 months, MMD remained at 9 (SD: 7), and MHD stabilized at 16 (SD: 11), reflecting sustained reductions of 8 (SD: 7) MMD and 8 (SD: 9) MHD. By 12 months, the mean MMD decreased further to 7 (SD: 7), and the MHD to 13 (SD: 10), representing reductions of 9 (SD: 7) MMD and 11 (SD: 9) MHD, respectively. These results are included in Table 3 and Table Supplementary 1.

**Table 3:** Effectiveness in patients with migraine under CGRP-mAbs and immunomodulatory treatments

Effectiveness	*n* = *89*
Reduction in MMD at 3 months, mean (SD)	8 (8)
Reduction in MHD at 3 months, mean (SD)	9 (9)
Reduction in MMD at 6 months, mean (SD)	8 (7)
Reduction in MHD at 6 months, mean (SD)	8 (9)
Reduction in MMD at 9 months, mean (SD)	9 (7)
Reduction in MHD at 9 months, mean (SD)	10 (9)
Reduction in MMD at 12 months, mean (SD)	9 (7)
Reduction in MHD at 12 months, mean (SD)	11 (9)
50% response in MMD at 3 months, *n* (%)	36 (43%)
50% response in MHD at 3 months, *n* (%)	31 (36%)
50% response in MMD at 6 months, *n* (%)	33 (49%)
50% response in MHD at 6 months, *n* (%)	26 (37%)
50% response in MMD at 9 months, *n* (%)	23 (55%)
50% response in MHD at 9 months, *n* (%)	21 (46%)
50% response in MMD at 12 months, *n* (%)	27 (61%)
50% response in MHD at 12 months, *n* (%)	23 (52%)

MHD, monthly headache days; MMD: monthly migraine days

The proportion of patients achieving a 50% reduction in MMD increased steadily over time, with 43% at 3 months, 49% at 6 months, 55% at 9 months, and 61% at 12 months. Similarly, the percentage achieving a 50% reduction in MHD rose from 36% at 3 months to 37% at 6 months, 46% at 9 months, and 52% at 12 months as shown in Table 3. Regarding effectiveness between patients receiving corticosteroids and patents with other immunomodulatory treatments, both groups showed significant changes in monthly migraine days (MMD) and monthly headache days (MHD) at 3, 6, 9, and 12 months compared to baseline, and patients receiving corticosteroids exhibited significantly smaller mean reductions in MMD and MHD at 3 and 6 months (Table Supplementary 2) without differences observed between groups in the proportion of patients achieving a ≥ 50% reduction in MMD or MHD. Moreover, there were no significant differences in ≥ 50% response rates at 3, 6, 9, and 12 months, nor in the reduction of monthly migraine days (MMD) or monthly headache days (MHD), between patients with and without other concomitant preventive treatments combined with immunomodulatory treatment at the initiation of anti-CGRP treatment.

### Safety in patients under CGRP-mAbs therapies with immunomodulatory treatments

The safety and tolerability profile of CGRP-mAbs with immunological treatments was assessed in 89 patients, revealing that 30% (27/89) experienced adverse events. The most commonly treatment-reported adverse events (TEAEs) included constipation, occurring in 20% (18/89), and dizziness, reported by 11% (10/89). Injection site reactions were observed in 7% (6/89) patients. Less frequently reported events included fatigue, headache, fever, HBP, and stroke, each occurring in 1% (1/89) of patients. The patient who experienced a stroke had ulcerative colitis and was undergoing anti-IL-17 treatment in concomitance with CGRP-mAbs. TEAEs are included in Table 4.

**Table 4:** Treatment emerging adverse events in patients with anti-CGRP-mAbs in combination with other immunomodulatory treatments

Treatment emerging adverse events	*n* = *89*
Adverse events, *n* (%)	27 (30%)
Constipation,* n* (%)	18 (20%)
Dizziness, *n* (%)	10 (11%)
Injection site reaction, *n* (%)	6 (7%)
Fatigue, *n* (%)	1 (1%)
Headache, *n* (%)	1 (1%)
Fever, *n* (%)	1 (1%)
High blood pressure, *n* (%)	1 (1%)
Stroke, *n* (%)	1 (1%)

### Characteristics of patients with autoimmune disease activation under CGRP-mAbs therapy

Among the nine patients experiencing autoimmune disease activation while on CGRP-mAbs and immunological treatment, key demographic and clinical characteristics are included in Table 5. All nine patients were women, with a mean age of 44 years (SD 13). Vascular risk factors were relatively uncommon, with one patient each reporting high blood pressure, dyslipidemia, and active smoking (11%), while none reported diabetes or alcohol consumption. Comorbid psychiatric conditions were notably prevalent, with anxiety and depression affecting 78% of the cohort, and insomnia present in 22%.

**Table 5:** Description of the patients under CGRP-mAbs with autoimmune disease activation

Variables	*n* = *9*
Age, years (SD)	44 (13)
Sex, female (%)	9 (100%)
Vascular risk factors	
High blood pressure, *n* (%)	1 (11%)
Dyslipidemia, *n* (%)	1 (11%)
Diabetes mellitus, *n* (%)	0 (0%)
Active smoking, *n* (%)	1 (11%)
Alcoholic consumption, *n* (%)	0 (0%)
Other comorbidities	
Anxiety, *n* (%)	7 (78%)
Depression, *n* (%)	7 (78%)
Insomnia, *n* (%)	2 (22%)
Age of migraine onset, mean (SD)	23 (11)
Chronic migraine, *n* (%)	7 (78%)
Migraine aura,* n* (%)	5 (56%)
Time with migraine, years (SD)	21 (16)
Time with chronic migraine, months (SD)	9 (6)
Medication overuse, *n* (%)	5 (56%)
Number of prior preventive treatments, median (IQR)	5 (4)
CGRP-mAbs plus immunomodulatory treatment duration, mean in months (IQR)	12 (18)
Autoimmune disease (AD) activation, *n* (%)	
Clinical, *n* (%)	8 (89%)
Radiological,* n* (%)	1 (11%)
Laboratory, *n* (%)	0 (0%)
Autoimmune disease type	
Neurological, *n* (%)	1
Rheumatological, *n* (%)	5
Gastrointestinal, *n* (%)	1
Dermatological, *n* (%)	1
Asthma and other allergies, *n* (%)	1
Immunosuppressants, *n* (%)	8 (89%)
Immunomodulatory therapies, *n* (%)	5 (55%)
Both, *n* (%)	4 (44%)
Baseline C-reactive protein	
Normal,* n* (%)	3 (60%)
Elevated,* n* (%)	2 (40%)
Baseline rheumatoid factor	
Normal, *n* (%)	2 (40%)
Elevated, *n* (%)	3 (60%)
Baseline VSG	
Normal, *n* (%)	3 (60%)
Elevated, *n* (%)	2 (40%)
Baseline lymphopenia	
No lymphopenia, *n* (%)	3 (60%)
Lymphopenia grade 1, *n* (%)	2 (40%)
Lymphopenia grade 2, *n* (%)	0 (0%)
Effectiveness of anti-CGRP therapies	
Reduction in MHD at 3 months, mean (SD)	11 (9)
Reduction in MMD at 3 months, mean (SD)	9 (8)
Reduction in MHD at 6 months, mean (SD)	14 (8)
Reduction in MMD at 6 months, mean (SD)	9 (6)
Reduction in MHD at 9 months, mean (SD)	14 (8)
Reduction in MMD at 9 months, mean (SD)	9 (5)
50% response rate in MMD at 3 months, *n* (%)	5 (56%)
50% response rate in MHD at 3 months, *n* (%)	4 (44%)
50% response rate in MMD at 6 months, *n* (%)	5 (56%)
50% response rate in MHD at 6 months, *n* (%)	5 (56%)
50% response rate in MMD at 9 months, *n* (%)	3 (33%)
50% response rate in MHD at 9 months, *n* (%)	3 (33%)
TEAEs, *n* (%)	5 (56%)
Dizziness, *n* (%)	3 (33%)
Constipation, *n* (%)	5 (56%)

SD, standard deviation; TEAEs, treatment emerging adverse events; IQR, interquartile range; MHD, monthly headache days; MMD, monthly migraine days

Migraine onset occurred at a mean age of 23 years (SD 11), and most patients (78%) suffered from CM, with 56% also experiencing MA and medication overuse. Patients had tried a median of five prior preventive treatments (IQR 4), and the combination of CGRP-mAbs with immunological treatments had been administered for an average of 12 months (IQR 18).

Autoimmune disease activation was primarily clinical. Rheumatological conditions were the most common type of autoimmune disease (56%), followed by neurological, gastrointestinal, dermatological, and allergic conditions. Immunological therapies were used by five patients. Baseline inflammatory markers, including CRP, RF, and VSG, were elevated in 40% of patients, while 40% also had grade 1 lymphopenia at baseline.

Despite these complexities, CGRP-mAbs demonstrated notable effectiveness, with reductions in MMD and MHD at 3, 6, 9, and 12 months. By 12 months, a 50% response rate was observed in 67% of patients for MMD and 44% for MHD. However, TEAEs were reported in 56% of the cohort, including dizziness (33%) and constipation (56%).

### Exploratory differences between patients with and without autoimmune disease activation

Anxiety [31 (39%) vs 7 (78%); *P* = 0.036] and migraine aura [18 (23%) vs 5 (56%; *P* = 0.049] were more prevalent among patients with immunological treatment that experienced a activation in their autoimmune disorder. Conversely, some vascular risk factors, including high blood pressure and dyslipidemia did not show differences between the groups (Table Supplementary 3).

Among the analyzed laboratory markers available at baseline and after CGRP-mAbs therapy start, baseline lymphopenia demonstrated a statistically significant difference between patients receiving CGRP-mAbs with immunomodulatory treatment (*P* = 0.049), with higher percentage of Lymphopenia grade 1 among patients with autoimmune diseases, while no statistically significant differences were observed for baseline VSG (*P* = 0.149), baseline CRP (*P* = 0.091) and baseline RF was no difference in those with autoimmune disease activation (Table Supplementary 4).

**Fig. 1:**
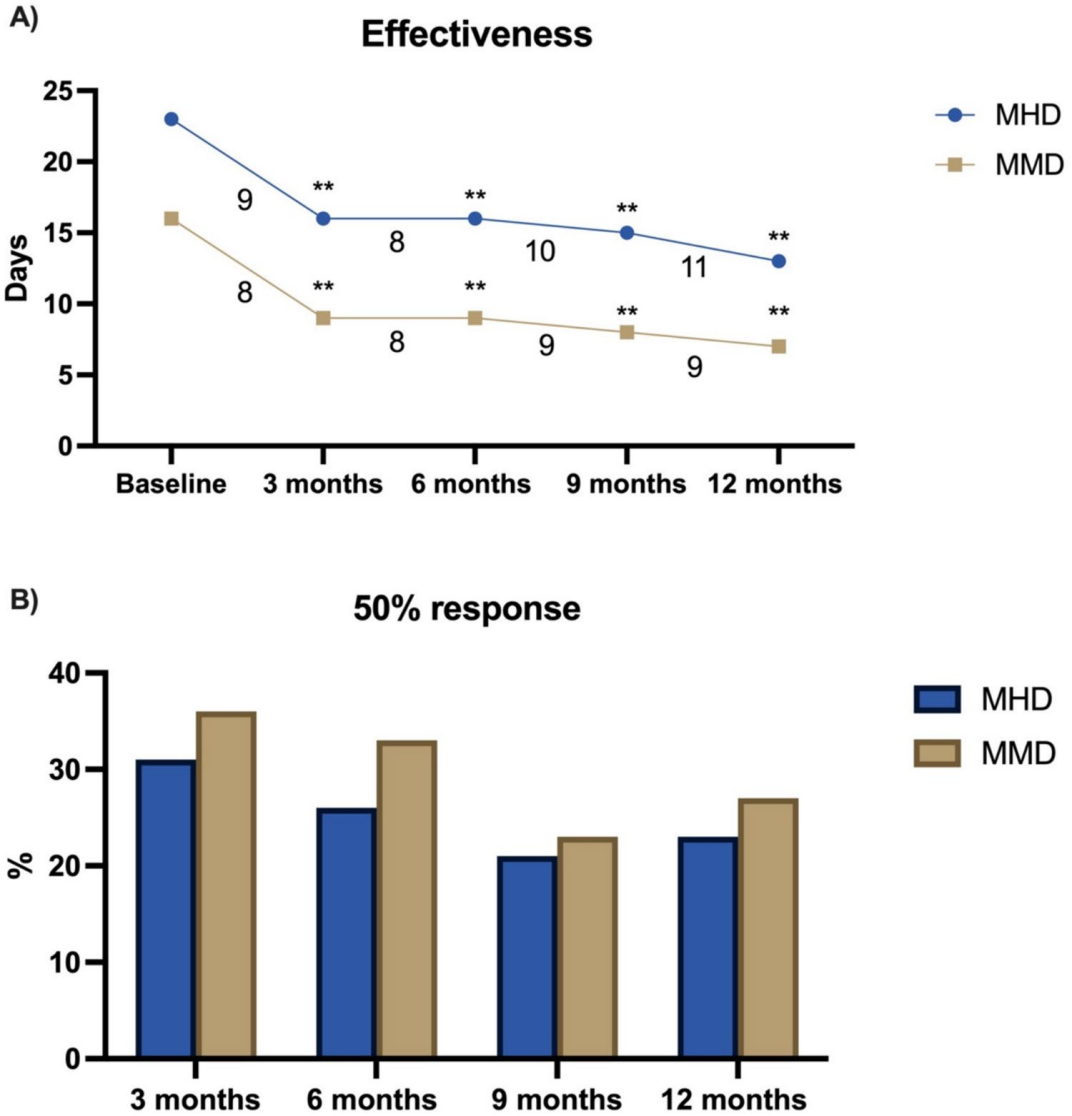
Effectiveness of anti-CGRP therapies in concomitance with immunological treatments in patients with migraine. **A** Monthly headache days (MHD) and monthly migraine days (MMD) at baseline and across follow-up, including absolute reductions. **B** Proportion of patients achieving a ≥ 50% response rate in MHD and MMD over time. MHD: monthly headache days, MMD: monthly migraine days; *: *p* < 0.05; **: *p* < 0.001

Regarding effectiveness in patients receiving CGRP-mAbs therapies with autoimmune disorders (Fig. 1), patients with autoimmune disease under immunological treatment that experienced autoimmune disease activation significantly showed fewer MHD at 6 months compared to those without it (mean 8.75 vs. 16.6; *P* = 0.015) with a similar outcome in both MHD and MMD in other time points nor reduction or response rate as included in Table Supplementary 5 and 6.

Regarding the presence of TEAEs, the overall presence of adverse events was similar between the two groups. When evaluating the different TEAEs separately, injection site reactions were more prevalent among patients with autoimmune disease activation (Table supplementary 7 and 8).

## Discussion

This study examines the clinical and demographic characteristics, as well as the effectiveness and safety of CGRP-mAbs therapies when combined with immunological treatments in patients with migraine and concomitant autoimmune diseases. The findings highlight particularly the favorable safety profile of monoclonal antibody therapies concomitant to mAbs therapies and the effectiveness of the concomitant treatment in patients with migraine and autoimmune disorders. Moreover, this study highlights differences between patients receiving CGRP-mAbs therapies with and without autoimmune diseases activation further adding to the scarce literature on this topic.

### Demographic and clinical characteristics

The demographic and clinical characteristics of the study cohort closely resemble those typically observed in the migraine population, characterized by a higher proportion of women; moreover, we found predominantly patients with chronic migraine—the primary population in which receiving CGRP-mAbs therapies have been introduced [19]. The mean age of the cohort, 44 years (SD 13), is consistent with established epidemiological patterns of migraine, with onset often occurring in early adulthood [4]. Furthermore, the cohort’s mean age of migraine onset, 23 years (SD 11), corresponds with the early onset trends reported in migraine epidemiology [4, 20].

While vascular risk factors were relatively uncommon—only one patient each reported HBP, DL, or active smoking (11%), and none reported DM or alcohol consumption—this aligns with findings that vascular comorbidities are not dominant contributors to migraine in younger and middle-aged populations [21, 22]. However, the notable prevalence of mood disorder comorbidities, particularly anxiety and depression (78%), is strikingly consistent with the literature on migraine, reflecting the well-documented association between migraine and psychiatric comorbidities [23, 24]. Insomnia, which affects 22% of this cohort, further emphasizes the multidimensional burden of the disease [25]. Moreover, a particularly notable finding was the high prevalence of medication overuse, affecting 56% of patients, a common complicating factor in CM, particularly among individuals seeking care at specialized headache centers [26, 27]. Furthermore, the cohort had tried a median of five prior preventive treatments, suggesting substantial therapeutic resistance, a hallmark of resistant CM populations [28]. These findings underscore the complexity of treating this subgroup of patients, particularly in the context of autoimmune comorbidities.

The therapeutic strategy combining CGRP-mAbs with immunosuppressants or immunomodulatory treatments for an average duration of 12 months gives valuable information of the treatment combination in our cohort.

### Laboratory markers

The baseline findings in patients undergoing CGRP-mAbs therapies alongside immunological treatments revealed predominantly normal inflammatory and hematological markers, such as CRP, RF, and VSG and a notable subset exhibiting elevated inflammatory marker levels. In addition, lymphopenia was observed in 8% of the patients, a recognized side effect, particularly with certain chemotherapies and immunotherapies [29]. Although CGRP-mAbs therapies are generally associated with a favorable safety profile in the existing literature [13], rare inflammatory complications have been reported in isolated case studies, suggesting the need for further investigation into potential pro-inflammatory responses in select individuals [15]. However, these findings should be interpreted with caution given the limited number of cases and the overall high tolerability observed in broader populations.

In our cohort, while most patients did not exhibit significant baseline abnormalities, the presence of elevated inflammatory markers and lymphopenia at baseline in a subset underscores the importance of individualized patient monitoring. These deviations may reflect underlying subclinical processes or predispositions that could influence therapeutic response or long-term prognosis. Further research is warranted to elucidate the clinical significance of these findings and to optimize management strategies for patients receiving CGRP-mAbs therapies in conjunction with other immunosuppressant and immunomodulatory treatments.

### Autoimmune disease activation in patients under CGRP-mAbs

Regarding potential clinical differences between patients with autoimmune diseases undergoing CGRP-mAbs therapies who experienced autoimmune disease activation versus those who did not, we observed a higher prevalence of anxiety among patients whose autoimmune disease worsened. This finding aligns with existing literature suggesting that inflammatory processes and the burden of chronic illness are closely linked. It is possible that elevated inflammatory levels in patients with more pronounced mood disorders could contribute to this relationship [30].

In addition, a higher percentage of patients with MA were observed among those who experienced autoimmune disease activation. This finding suggests a potential interaction between an inflammatory environment in patients with MA and autoimmune disorders, wherein CGRP levels might play a pivotal role. While rare cases have raised concerns about potential interactions between CGRP-mAbs therapies and autoimmune disease activity, particularly in patients with coexisting migraine with aura (MA), current evidence is limited and largely based on small observational samples [31, 32]. Further research is needed to clarify any potential impact on autoimmune disease progression.

Patients receiving concomitant immunomodulatory treatments demonstrated improvements in monthly migraine days (MMD) and monthly headache days (MHD) over time compared to baseline. Similar findings have been previously reported in patients with migraine receiving other concomitant monoclonal antibodies, as well as in those with multiple sclerosis [33, 34]. In addition, patients receiving corticosteroids showed a trend toward fewer monthly headache days (MHD) and monthly migraine days (MMD), with a statistically significant reduction observed at 6 months. Although corticosteroids are not established as preventive treatments for migraine, their chronic use—typically in the context of severe or active autoimmune disorders—may exert analgesic effects that could partially contribute to the observed response to anti-CGRP therapies [35]. However, our findings suggest that any potential benefit may be offset by the underlying autoimmune disease activity and/or systemic inflammation, which may explain the slightly attenuated treatment response observed in this subgroup. This potential confounding effect and its implications warrant further investigation. Overall, the use of anti-CGRP therapies in combination with other immunosuppressants appears to be effective in patients requiring these medications for the management of their autoimmune disorders. Although all patients were receiving immunomodulatory treatments, those who experienced autoimmune disease activation showed a trend toward a more pronounced reduction. These findings may be influenced by the intensification of immunosuppressive therapy and highlight the interplay between inflammation, autoimmune activity, and migraine management, calling for integrated approaches combining immunomodulatory and migraine-specific therapies. This reinforces the potential utility of combining immunomodulatory therapies with CGRP-mAbs treatments to optimize outcomes in autoimmune populations.

Moreover, the combination of CGRP-mAbs therapies with other immunological treatments (Table Sup 7) is overall safe with a similar percentage of TEAEs. Patients with autoimmune disease activation showed a higher use of immunosuppressants and a tendency towards higher use of corticosteroids indicating a possible uncontrolled disease or severe or resistant cases. This trend warrants further exploration, as long-term treatment might lead to adverse effects and may not effectively modify disease progression in some cases. In contrast, monoclonal antibody therapies were used similarly in both groups suggesting they are relatively safe and not strongly associated with autoimmune disease activation. These targeted therapies, including anti-TNF, anti-IL-6, and anti-CD20 agents, are essential in autoimmune disease management and may provide disease stabilization without significant risk of autoimmune disease activation.

The connection between inflammatory, immunological, and vascular processes shared by migraine and autoimmune diseases has become increasingly evident [36]. Similarly, laboratory markers such as CGRP elevation have been linked not only to migraine but also to hyperinflammation in conditions like COVID-19 [37]. In addition, a study published in 2023 study suggested that immuno-rheumatological comorbidities might negatively affect the response to CGRP-mAbs treatments [38]. However, previous findings by González-Martínez et al. (2022) in a cohort of patients with MS treated with disease-modifying therapies (DMT) showed that CGRP-mAbs in combination with multiple sclerosis DMT are effective and did not increase outbreaks or infections after 18 months, aligning with our study’s lower rates of autoimmune disease activation and TEAEs. Although constipation was the most common adverse event, only 5% of patients discontinued treatment due to side effects, suggesting good overall tolerance. Moreover, injection site reactions were more frequently observed in patients on concomitant immunomodulatory therapies—common in neuroimmunological conditions such as multiple sclerosis—likely due to localized immune priming. The administration of multiple subcutaneous treatments, particularly when cumulative injection volumes exceed 1.5 mL, may lead to increased tissue distension, delayed absorption, and amplified local inflammatory responses, contributing to higher injection site reaction rates in this population [39, 40].

### Limitations and strengths

The study’s limitations include a small sample size, particularly in the autoimmune disease activation group, reducing statistical power and generalizability of the exploratory comparison. Short follow-up periods may underestimate long-term safety and efficacy, highlighting the need for extended longitudinal studies. Since 90% of the cohort were women, the results may not be fully generalizable to male patients with similar conditions. However, this study provides valuable insights into the use of CGRP-mAbs combined with immunosuppressants or immunomodulatory treatments in patients with migraine and autoimmune diseases, a population that is underrepresented in existing research. The focus on real-world outcomes, including treatment safety, effectiveness, and disease activation, adds clinical relevance for clinical practice.

## Conclusions

This study provides one of the first systematic evaluations of the concurrent use of CGRP-mAbs and immunological treatments, offering a foundation for future clinical guidelines in patients with neurological and autoimmune conditions. The results show that the combination of CGRP-mAbs therapies with immunological treatments appears effective and safe, offering a promising option for patients with resistant and refractory CM and autoimmune comorbidities. However, the observed trend toward corticosteroid use in active cases calls for cautious, individualized approaches to minimize potential long-term risks. Monoclonal antibody therapies remain a cornerstone of treatment, providing disease stabilization without a significant increase in adverse outcomes. This study also underscores the importance of individualized approaches in managing migraine patients with autoimmune diseases. The overall safety and efficacy of CGRP-mAbs therapies support their continued use in these populations. Future research should focus on identifying biomarkers of response to CGRP-mAbs therapies in autoimmune populations and exploring the mechanisms underlying the interaction between CGRP inhibition and immunomodulatory treatments. Larger cohorts and longer follow-ups are needed to confirm these findings and refine treatment strategies for this complex patient population.

## Bullet points

- CGRP-mAbs therapies combined with immunological treatments show favorable effectiveness and safety in autoimmune disease patients.
- Patients with chronic migraine and autoimmune disorders under CGRP-mAbs and immunomodulatory treatment exhibited high comorbid anxiety (78%) and medication overuse (56%).
- Elevated inflammatory markers and lymphopenia in a subset of patients underscore the need for individualized monitoring.
- Autoimmune disease activation linked to anxiety and migraine aura in patients with migraine receiving CGRP-mAbs and immunological treatments needs further attention.
- These findings support integrated migraine and autoimmune treatment management to improve disease control.

## Supplementary Information

Below is the link to the electronic supplementary material.

Supplementary file1 (DOCX 28 KB)

## Data Availability

The datasets used and/or analyzed during the current study are available from the corresponding author on reasonable request.

## Abbreviations

MMD: Monthly migraine days
MHD: Monthly headache days
CGRP: Calcitonin gene-related peptide
CGRP-mAbs: Anti-CGRP monoclonal antibodies
TEAEs: Treatment-emergent adverse events
HBP: High blood pressure
DL: Dyslipidemia (DL)
DM: Diabetes mellitus
CM: Chronic migraine
EM: Episodic migraine
MA: Migraine aura
CRP: C-reactive protein (CRP)
RF: Rheumatoid factor (RF)
VSG: Erythrocyte sedimentation rates
