# Supplementary material for: Concomitant anti-CGRP and immunomodulatory treatments in patients with migraine: towards integrated management strategies
Source: J Neurol. 2025 Jun 3;272(6):443. doi: 10.1007/s00415-025-13177-y (PMC12134006; doi:10.1007/s00415-025-13177-y)
Supplement: Supplementary file 1 — Supplementary file1 (DOCX 28 KB) [file 415_2025_13177_MOESM1_ESM.docx]

**Table Supplementary 1. Effectiveness in patients with migraine under CGRP-mAbs and immunological treatments.**

| **Effectiveness** | ***n=89*** |
| --- | --- |
| MMD at baseline, mean (SD) | 16 (7) |
| MHD at baseline, mean (SD) | 23 (8) |
| MMD at 3 months, mean (SD) | 9 (7) |
| MHD at 3 months, mean (SD) | 16 (10) |
| MMD at 6 months, mean (SD) | 9 (7) |
| MHD at 6 months, mean (SD) | 16 (11) |
| MMD at 9 months, mean (SD) | 8 (7) |
| MHD at 9 months, mean (SD) | 15 (11) |
| MMD at 12 months, mean (SD) | 7 (7) |
| MHD at 12 months, mean (SD) | 13 (10) |

MHD: Monthly Headache Days, MMD: Monthly Migraine Days.

**Table Supplementary 2. Reduction in MMD and MHD in patients under CGRP-mAbs corticosteroids versus other immunomodulatory treatments.**

| **Demographic variables** | ***Anti-CGRP mAbs + immunomodulatory treatments***  ***N=70*** | ***Anti-CGRP mAbs + corticosteroids***  ***N=18*** | ***P-value*** |
| --- | --- | --- | --- |
| Reduction in MMD at 3m, mean (SD) | 8.52 (8.13) | 4.36 (4.41) | 0.011* |
| Reduction in MHD at 3m, mean (SD) | 10.1 (9.57) | 3.57 (4.99) | 0.001* |
| Reduction in MMD at 6m, mean (SD) | 9.53 (7.52) | 4.46 (4.33) | 0.004* |
| Reduction in MHD at 6m, mean (SD) | 9.63 (9.13) | 4.00 (6.18) | 0.014* |
| Reduction in MMD at 9m, mean (SD) | 10.9 (7.35) | 4.73 (4.31) | 0.003* |
| Reduction in MHD at 9m, mean (SD) | 12.2 (9.70) | 4.50 (5.66) | 0.003* |
| Reduction in MMD at 12m, mean (SD) | 9.81 (7.10) | 6.67 (4.74) | 0.135 |
| Reduction in MHD at 12m, mean (SD) | 8.52 (8.13) | 4.36 (4.41) | 0.011* |

m: months; MHD: Monthly Headache Days, MMD: Monthly Migraine Days.

**Table Supplementary 3. Comparison of the demographic characteristics of patients under CGRP-mAbs with and without autoimmune disease activation.**

| **Demographic variables** | ***Inactive autoimmune disease***  ***n=79*** | ***Active autoimmune disease***  ***n=9*** | ***P-value*** |
| --- | --- | --- | --- |
| **Age, years (SD), min-max** | 50 (10) | 44 (13) | 0.206 |
| **Sex, female (%)** | 70 (89 %) | 9 (100 %) | 0.589 |
| **Vascular risk factors** |  |  |  |
| Hight Blood Pressure, *n* (%) | 10 (13 %) | 1 (11%) | 0.999 |
| Dyslipidemia, *n* (%) | 12 (15 %) | 1 (11%) | 0.999 |
| Diabetes Mellitus, *n* (%) | 3 (4 %) | 0 (0 %) | 0.999 |
| Active smoking, *n* (%) | 16 (20 %) | 1 (11 %) | 0.999 |
| Alcoholic consumption, *n* (%) | 0 (0 %) | 0 (0 %) | -- |
| **Other comorbidities** |  |  |  |
| **Anxiety, *n* (%)** | **31 (39 %)** | **7 (78 %)** | **0.036*** |
| Depression, *n* (%) | 37 (47 %) | 7 (78 %) | 0.157 |
| Insomnia, *n* (%) | 24 (30 %) | 2 (22 %) | 0.999 |
| **Age of migraine onset, median (IQR)** | 20 (13) | 25 (11) | 0.515 |
| **Chronic migraine, *n* (%)** | 68 (86 %) | 7 (78 %) | 0.616 |
| **Migraine with aura, *n* (%)** | **18 (23 %)** | **5 (56 %)** | **0.049*** |
| **Time with migraine, years (SD)** | 27 (12) | 21.3 (16) | 0.315 |
| **Time with chronic migraine, months (SD)** | 11 (10) | 8.85 (6) | 0.307 |
| **Medication overuse, *n* (%)** | 54 (70%) | 5 (56%) | 0.609 |
| **Age of AD onset, median (IQR)** | 38 (18) | 39 (5) | 0.370 |
| **Number of prior preventive treatments, median (IQR)** | 5 (4) | 5 (1) | 0.761 |
| **AntiCGRP plus Immunomodulatory treatment duration, median in months (IQR)** | 12 (18) | 12 (0) | 0.874 |

SD: Standard Deviation; TEAEs: Treatment Emerging Adverse Events; IQR: Interquartile Range; *: *p*<0.05

**Table Supplementary 4. Comparison of the laboratory baseline variables in patients under CGRP-mAbs** **with and without autoimmune disease activation.**

| **Laboratory markers** | ***Inactive autoimmune disease***  ***n=58*** | | ***Active autoimmune disease***  ***n=9*** | ***P-value*** |
| --- | --- | --- | --- | --- |
| **Baseline C-reactive protein** | |  |  | 0.091 |
| Normal, *n* (%) | | 53 (91 %) | 3 (60 %) |  |
| Elevated, *n* (%) | | 5 (9 %) | 2 (40 %) |  |
| **Baseline Rheumatoid Factor** | |  |  | 0.064 |
| Normal, *n* (%) | | 37 (82 %) | 2 (40 %) |  |
| Elevated, *n* (%) | | 8 (18 %) | 3 (60 %) |  |
| **Baseline VSG** | |  |  | 0.149 |
| Normal, *n* (%) | | 44 (88 %) | 3 (60 %) |  |
| Elevated, *n* (%) | | 6 (12 %) | 2 (40 %) |  |
| **Baseline lymphopenia** | |  |  | **0.049*** |
| **No lymphopenia, *n* (%)** | | **53 (95 %)** | **3 (60 %)** |  |
| **Lymphopenia grade 1, *n* (%)** | | **2 (4 %)** | **2 (40 %)** |  |
| **Lymphopenia grade 2, *n* (%)** | | **1 (2 %)** | **0 (0 %)** |  |

VSG: Erythrocyte Sedimentation Rate; Lymphopenia Grade 1: 800–1,000 cells/μL; Lymphopenia Grade 2: 500–799 cells/μL; *: *p*<0.05.

**Table Supplementary 5. Effectiveness in patients with migraine under CGRP-mAbs and immunological treatment with and without autoimmune disease activation.**

| Effectiveness | ***Inactive autoimmune disease***  ***n=79*** | ***Active autoimmune disease***  ***n=9*** | ***P-value*** |
| --- | --- | --- | --- |
| MMD at baseline, mean (SD) | 23 (8) | 22 (7) | 0.651 |
| MHD at baseline, mean (SD) | 16 (7) | 15 (6) | 0.653 |
| MHD at 3 months, mean (SD) | 16 (11) | 12 (7) | 0.138 |
| MMD at 3 months, mean (SD) | 9 (8) | 7 (5) | 0.333 |
| **MHD at 6 months, mean (SD)** | **17 (11)** | **9 (7)** | **0.015*** |
| MMD at 6 months, mean (SD) | 9 (8) | 6 (4) | 0.132 |
| MHD at 9 months, mean (SD) | 16 (12) | 11 (6) | 0.163 |
| MMD at 9 months, mean (SD) | 8 (7) | 7 (3) | 0.738 |
| MHD at 12 months, mean (SD) | 14 (11) | 10 (7) | 0.221 |
| MMD at 12 months, mean (SD) | 8 (7) | 5 (3) | 0.053 |

MHD: Monthly Headache Days, MMD: Monthly Migraine Days; *p*<0.05; *: *p*<0.05.

**Table Supplementary 6. Effectiveness in patients under CGRP-mAbs and immunological treatments with and without autoimmune disease activation.**

| **Effectiveness** | ***Inactive autoimmune disease***  ***n=79*** | ***Active autoimmune disease***  ***n=9*** | ***P-value*** |
| --- | --- | --- | --- |
| Reduction in MMD at 3m, mean (SD) | 8 (8) | 9 (8) | 0.737 |
| Reduction in MHD at 3m, mean (SD) | 8 (9.38) | 11 (9) | 0.557 |
| Reduction in MMD at 6m, mean (SD) | 8 (7.47) | 9 (6) | 0.826 |
| Reduction in MHD at 6m, mean (SD) | 8 (8.83) | 14 (8) | 0.051 |
| Reduction in MMD at 9m, mean (SD) | 9 (7.66) | 9 (5) | 0.824 |
| Reduction in MHD at 9m, mean (SD) | 9 (9.64) | 14 (8) | 0.225 |
| Reduction in MMD at 12m, mean (SD) | 9 (6.95) | 11 (6) | 0.304 |
| Reduction in MHD at 12m, mean (SD) | 10 (8.89) | 15 (8) | 0.129 |
| 50% response in MMD at 3 months, *n* (%) | 30 (41 %) | 5 (56 %) | 0.486 |
| 50% response in MHD at 3 months, *n* (%) | 26 (35 %) | 4 (44 %) | 0.715 |
| 50% response in MMD at 6 months, *n* (%) | 27 (46 %) | 5 (62 %) | 0.464 |
| 50% response in MHD at 6 months, *n* (%) | 20 (32 %) | 5 (62 %) | 0.124 |
| 50% response in MMD at 9 months, *n* (%) | 19 (56 %) | 3 (43%) | 0.685 |
| 50% response in MHD at 9 months, *n* (%) | 17 (45 %) | 3 (43 %) | 0.999 |
| 50% response in MMD at 12 months, *n* (%) | 20 (56 %) | 6 (85 %) | 0.215 |
| 50% response in MHD at 12 months, *n* (%) | 18 (50 %) | 4 (57 %) | 0.999 |

m: months; MHD: Monthly Headache Days, MMD: Monthly Migraine Days.

**Table Supplementary 7. Adverse Events in patients under CGRP-mAbs** **and immunological treatment with and without autoimmune disease activation.**

| **Adverse Events** | ***Inactive autoimmune disease***  ***n=79*** | ***Active autoimmune disease***  ***n=9*** | ***P-value*** |
| --- | --- | --- | --- |
| **Adverse events, n (%)** | 22 (28 %) | 5 (56 %) | **0.126** |
| Dizziness, *n* (%) | 7 (9 %) | 3 (33 %) | 0.062 |
| **Constipation, *n* (%)** | **13 (16 %)** | **5 (56 %)** | **0.016*** |
| Injection site reaction, *n* (%) | 6 (8 %) | 0 (0 %) | 0.999 |
| Fatigue, *n* (%) | 1 (1 %) | 0 (0 %) | 0.999 |
| Headache, *n* (%) | 1 (1 %) | 0 (0 %) | 0.999 |
| Fever, *n* (%) | 1 (1 %) | 0 (0 %) | 0.999 |
| HBP, *n* (%) | 1 (1 %) | 0 (0 %) | 0.999 |
| Stroke, *n* (%) | 1 (1 %) | 0 (0 %) | 0.999 |

HBP: High Blood Pressure; *p*<0.05.

**Table Supplementary 8. Concomitant immunological treatments grouped by mechanisms of action in patients with migraine and autoimmune disorders under CGRP-mAbs.**

| **Concomitant treatments** | ***Inactive autoimmune disease***  ***n=79*** | ***Active autoimmune disease***  ***n=9*** | ***P-value*** |
| --- | --- | --- | --- |
| **Immunosuppressors, *n* (%)** | 47 (59.5%) | 8 (88.9%) | 0.145 |
| Immunomodulators, *n* (%) | 38 (48.1%) | 5 (55.6%) | 0.736 |
| **Both, *n* (%)** | **8 (10.1%)** | **4 (44.4%)** | **0.018*** |
| Amino salicylates, *n* (%) | 6 (7.59%) | 0 (0.00%) | 0.999 |
| Corticosteroids, *n* (%) | 14 (17.7%) | 4 (44.4%) | 0.080 |
| Antimetabolites, *n* (%) | 20 (25.3%) | 2 (22.2%) | 0.999 |
| Calcineurin inhibitors *n* (%) | 3 (3.80%) | 0 (0.00%) | 0.999 |
| Anti-JAK, *n* (%) | 1 (1.27%) | 1 (11.1%) | 0.999 |
| Anti-CD20, *n* (%) | 5 (6.33%) | 0 (0.00%) | 0.999 |
| IFN-B1b, *n* (%) | 1 (1.27%) | 0 (0.00%) | 0.999 |
| IFN-B1a, *n* (%) | 4 (5.06%) | 1 (11.1%) | 0.425 |
| Anti-IL6, IL-12, IL-17, IL-23 IL-5, *n* (%) | 9 (11.4%) | 0 (0.00%) | 0.999 |
| Anti-TNF, *n* (%) | 10 (12.7%) | 1 (11.1%) | 0.999 |
| B cell modulators, *n* (%) | 0 (0.00%) | 1 (11.1%) | 0.102 |
| Anti-IgE, *n* (%) | 1 (1.27%) | 0 (0.00%) | 0.999 |
| CD52 modulators, *n* (%) | 2 (2.53%) | 0 (0.00%) | 0.999 |
| Costimulation modulators, *n* (%) | 1 (1.27%) | 0 (0.00%) | 0.999 |
| Any monoclonal antibodies, *n* (%) | 21 (26.6%) | 2 (22.2%) | 0.999 |
| Immunoglobulins iv, *n* (%) | 1 (1.27%) | 0 (0.00%) | 0.999 |
| Other treatments, *n* (%) | 16 (20.3%) | 3 (33.3%) | 0.399 |

TNF: Tumor Necrosis Factor; IL: Interleukin; CD: Cluster of Differentiation; JAK: Janus Kinase. Other treatments include: Hydroxychloroquine, Montelukast, Siponimod, Dimethyl fumarate, Glatiramer acetate, Leflunomide. *p*<0.05.
